# *MIR172d* Is Required for Floral Organ Identity and Number in Tomato

**DOI:** 10.3390/ijms22094659

**Published:** 2021-04-28

**Authors:** Wanping Lin, Suresh Kumar Gupta, Tzahi Arazi, Ben Spitzer-Rimon

**Affiliations:** 1 Institute of Plant Sciences, Agricultural Research Organization—Volcani Institute, HaMaccabbim Road 68, Rishon LeZion 7505101, Israel; wanping.lin@mail.huji.ac.il (W.L.); suresh@volcani.agri.gov.il (S.K.G.); 2 Department of Plant Science, The Robert H. Smith Faculty of Agriculture, Food and Environment, The Hebrew University of Jerusalem, P.O. Box 12, Rehovot 7610001, Israel

**Keywords:** tomato, flower, miRNA, miR172, APETALA, CRISPR, Cas9

## Abstract

MicroRNA172 (miR172) functions as a central regulator of flowering time and flower development by post-transcriptional repression of APETALA2-LIKE transcription factors. In the model crop *Solanum lycopersicum* (tomato), the miR172 family is still poorly annotated and information about the functions of specific members is lacking. Here, de-novo prediction of tomato miR172 coding loci identified seven genes (*SlMIR172a-g*), that code for four unique species of miR172 (sly-miR172). During reproductive development, sly-miR172s are differentially expressed, with sly-miR172c and sly-miR172d being the most abundant members in developing flowers, and are predicted to guide the cleavage of eight APETALA2-LIKE transcription factors. By CRISPR-Cas9 co-targeting of *SlMIR172c* and *SlMIR172d* we have generated a battery of loss-of-function and hypomorphic mutants (*slmir172c-d^CR^*). The *slmir172c-d^CR^* plants developed normal shoot but their flowers displayed graded floral organ abnormalities. Whereas *slmir172c^CR^* loss-of-function caused only a slight greening of petals and stamens, hypomorphic and loss-of-function *slmir172d^CR^* alleles were associated with the conversion of petals and stamens to sepaloids, which were produced in excess. Interestingly, the degrees of floral organ identity alteration and proliferation were directly correlated with the reduction in sly-miR172d activity. These results suggest that sly-miR172d regulates in a dose-dependent manner floral organ identity and number, likely by negatively regulating its APETALA2-like targets.

## 1. Introduction

Plant microRNAs (miRNAs), which are coded by *MICRORNA* (*MIR*) genes, constitute a major class of endogenous small RNAs that trigger sequence-specific post-transcriptional repression of target mRNAs with high sequence complementarity [1]. Studies of plant miRNA biology established their critical roles in a wide range of developmental processes, stresses and nutrient homeostasis [2,3,4]. MiR172 is highly conserved in seed plants, but was not identified in more ancient plant clades such as lycopods and bryophytes [5]. The miR172 family was identified in at least 30 angiosperm species and contains up to 11 members (*Glycine max*) [6]. MiR172 targets mRNAs that encode euphyllophyte APETALA2 (euAP2) transcription factors, a lineage of the AP2/ethylene responsive factor (ERF) superfamily, by both transcript cleavage as well as by translational inhibition mechanisms [7,8]. All euAP2 are characterized by the presence of two AP2 domains and a downstream target site for miR172, and are further divided into TARGET OF EAT (TOE)- and AP2-type genes according to the similarity of their encoded proteins to AP2 or TOE proteins, respectively [7,9,10].

The conservation of the miR172/euAP2 regulatory module in the seed plant lineage suggests that it played a critical component in their evolution [10]. Indeed, the euAP2/miR172 regulatory module was found to be involved in central aspects of seed plant development [7,8,11]. The regulation of certain *euAP2* by miR172 is essential for the transition from vegetative growth to flowering. As the plant matures, miR156 is downregulated resulting in the upregulation of its target mRNAs, which encode transcription factors that belong to the SQUAMOSA PROMOTER BINDING PROTEIN-LIKE (SPL), that in turn activate the expression of *MIR172* genes [12]. As a result, the levels of the miR172 targets *AP2*, *TOE1*, *TOE2*, *SCHLAFMUTZE* (*SMZ*) and *SCHNARCHZAPFEN* (*SNZ*), which suppress the development of adult phase traits, are decreasing, to promote plant maturation and flowering [7,8,12,13,14]. Recently, a comprehensive functional analysis of *MIR172* genes in *Arabidopsis* has shown that under non-inductive photoperiod, floral transition is dependent on the downregulation of *TOE2* and *AP2* mainly by *MIR172d* and to a lesser degree by *MIR172a* and *MIR172b* at the shoot apical meristem. Under inductive photoperiod, *MIR172b*, which is the main family member expressed in leaves, together with *MIR172a* promote flowering most probably by targeting AP2-LIKE transcription factors that suppress the expression of the florigen-encoding *FLOWERING LOCUS T* in leaves [13,15,16,17,18].

The euAP2/miR172 regulatory module also plays important role in the regulation of floral organ identity. Perfect flower development requires the formation of four distinct organ types, two outer perianth whorls i.e., sepals and petals, and two reproductive inner whorls i.e., stamens and carpels. The specification of the floral domains was initially explained through the ABC model that was later expanded to the ABCDE model. According to the model, which was initially established in snapdragon and *Arabidopsis*, the A-function genes *AP1* and the miR172-regulated *AP2s* establish the identity of sepals in the outer whorl; and together with the B-function genes *APETALA3* (*AP3*) and *PISTILLATA* (*PI*) direct petal identity in the second whorl. Stamens development in the third whorl is governed by the B-function genes together with the C-function gene *AGAMOUS* (*AG*). The latter also governs carpel development in the fourth whorl as well as conferring floral meristem termination (also known as floral determinacy) [19,20,21,22]. A mutual antagonistic interaction of A- and C-function genes is one of the key components in the ABCDE model that derive accurate patterning of floral organ identity. In the *ag* mutant, expansion of A-function activity towards the flower inner whorls takes place resulting in the development of petals and sepals instead of stamens and carpels, respectively [19,20,21]. On the other hand, *AP2* loss-of-function led to the expansion of C-function activity to the outer whorls, resulting in flowers with no sepals and petals and instead carpelloid and stamen-like structures, respectively [11,21,23]. Consistently, in flowers that express miR172-resistant version of *AP2* as well as in flowers with reduced miR172 activity, the stamens were partially or completely converted into petals [24,25]. While the regulatory roles in flower development of the B- and C- function genes are conserved across various species, the roles of the *AP2s* A- function genes are diverged. This was demonstrated in petunia, in which the C- function activity is repressed mainly by the miR169 *BLIND* and to lesser extent by the TOE-type A- function gene *BLIND ENHANCER* (*PhBEN*). Moreover, *PhBEN* together with the AP2-type A- function genes *REPRESSOR OF B-FUNCTION* (*PhROBs*) repress B-function genes in the flower’s first whorl. Correspondingly, sepals were converted into petals in flowers of a *ben rob1 rob2* mutant [26,27].

Tomato (*Solanum lycopersicum*), is an important model crop plant, but at present information about the functions of its miRNAs is limited. Recently, the use of clustered regularly interspaced short palindromic repeats (CRISPR)-CRISPR associated protein9 (Cas9) to mutate specific tomato *MIR* genes provided insights into their specific regulatory roles in development [28]. In contrast to the comprehensive understanding of miR172 and individual *MIR172* gene functions in *Arabidopsis* [7,17,18], only two functional studies on tomato miR172 were published to date. These describe the ectopic expression of miR172 that resulted in the development of flowers with enlarged sepals showing sepal-to-petal transformation [29,30]. In the current study, we performed extensive bioinformatic analysis of public small RNA and degradome data to identify all transcriptionally active sly-miR172 coding loci in tomato and their possible target mRNAs, respectively. We then used CRISPR/Cas9 technology, to mutate *SlMIR172C* and *SlMIR172D*, which are abundant in developing flowers. By dissecting generated CRISPR mutants, we uncovered the requirement of *SlMIR172D* for tomato floral organ identity and number.

## 2. Results

### 2.1. The Tomato miR172 Family of miRNAs

**Figure 1:**
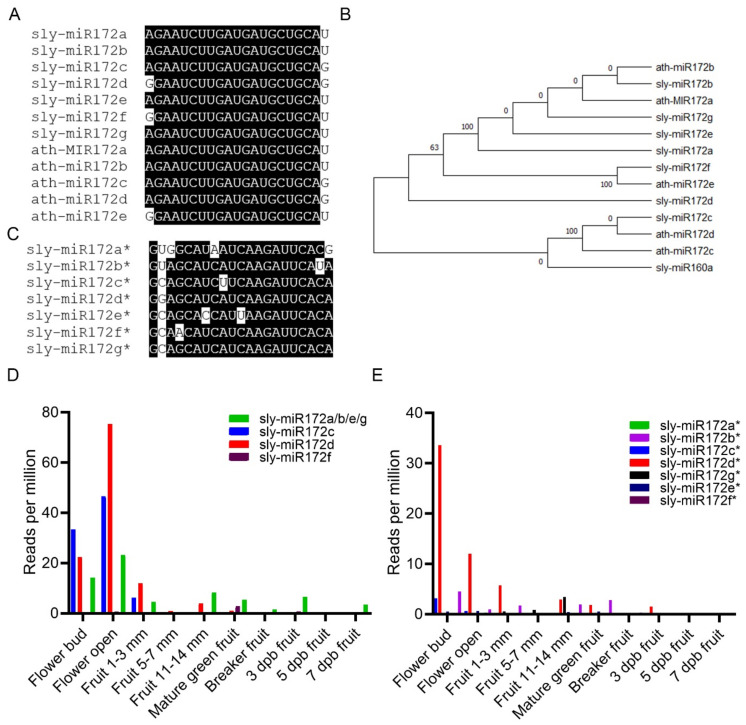
The tomato miR172 family. (**A**) Nucleotide sequence alignment of *Arabidopsis* (ath-miR172) and tomato (sly-miR172) miR172 family members. (**B**) A rooted phylogenetic tree based on the *Arabidopsis* and tomato miR172 family members. Sly-miR160a served as an outgroup. The tree was constructed using FastTree v2.1.8 with default parameters [34]. (**C**) Nucleotide sequence alignment of tomato miR172 members star strands. (**D**,**E**) Accumulation of sly-miR172 members mature (**D**) and star (**E**) strands in tomato cv. MicroTom anthesis flowers and pericarp of developing fruits as indicated. Mature sly-miR172a, sly-miR172b, sly-miR172e and sly-miR172g have identical sequences hence the single profile (sly-miR72a/b/e/g). The expression data was retrieved from the Tomato Functional Genomics Database (http://ted.bti.cornell.edu/, accessed on 1 January 2021). dpb-days post breaker.

**Figure A1:**
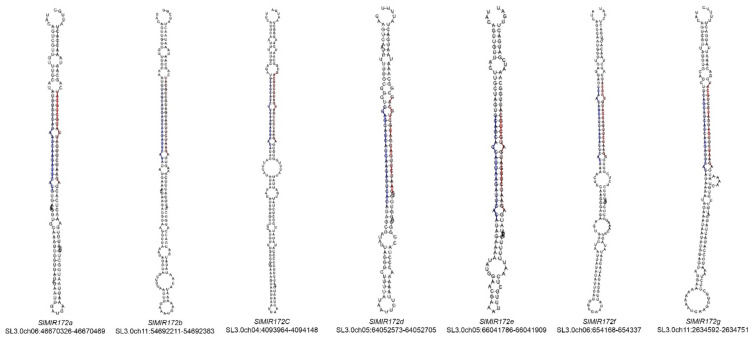
Hairpin secondary structures of sly-miR172 precursors as predicted by RNAfold (http://rna.tbi.univie.ac.at/cgi-bin/RNAWebSuite/RNAfold.cgi, accessed on 1 January 2021). The position of each precursor in the tomato genome (SL3.0) is indicated. Mature sly-miR172 and respective sly-miR172* sequences are marked by red and blue, respectively.

Several different tomato sly-miR172 sequences were previously identified via small RNA cloning from leaf, flower bud, and fruit designated sly-miR172a-d [31,32,33]. To identify all potential miR172 members in tomato, de-novo *MIR* loci prediction using high-volume 20–22 nt small RNA data, assembled from 178 small RNA public data sets representing various healthy and virus-infected vegetative as well as reproductive tissues (Table S1), was used to inform on all the transcriptionally active *MIR172* loci in the tomato genome. This analysis identified seven *SlMIR172* genes (Figure A1 and Table S2). Multiple sequence alignment and phylogenetic analysis of mature miR172 from tomato revealed that they are highly conserved except for their last and first nucleotides (Figure 1A). With the exception of sly-miR172d, the other miR172 family members have an identical *Arabidopsis* member as follows: sly-miR172a, sly-miR172b, sly-miR172e and sly-miR172g sequences are identical to ath-miR172a and ath-miR172b; sly-miR172c is identical to ath-miR172c and sly-miR172f is identical to ath-miR172e (Figure 1A,B). Conversely, multiple sequence alignment of the star strands (miR172*) of identified miRNAs indicated that they are less conserved than the miRNAs and each has a unique sequence (Figure 1C).

In Silico expression analysis of sly-miR172 and sly-miR172* members in MicroTom developing flowers and fruit pericarp indicated that the mature sly-miR172c and sly-miR172d species are more abundant in flower buds and anthesis flowers than the rest of the sly-miR172 members, whereas the identical sly-miR172a/b/e/g species, which is coded by *SlMIR172a*, *SlMIR172b*, *SlMIR172e* and *SlMIR172g*, is the most prevalent miR172 member in developing and ripening fruits (Figure 1D). Moreover, sly-miR172d* was more prevalent than other sly-miR172* in the flower buds, anthesis flowers and young immature fruits, implying that sly-miR172d is expressed at relatively high levels in these organs. Of the sly-miR172a/b/e/g star species, sly-miR172b* was the most abundant in flowers and fruits suggesting that *SlMIR172b* is the dominant contributor of sly-miR172a/b/e/g species in these tissues. These results suggest that *SlMIR172c* and *SlMIR172d* are predominant during flower development and fruit set.

### 2.2. Generation of SlMIR172c and SlMIR172d CRISPR Mutants

**Figure 2:**
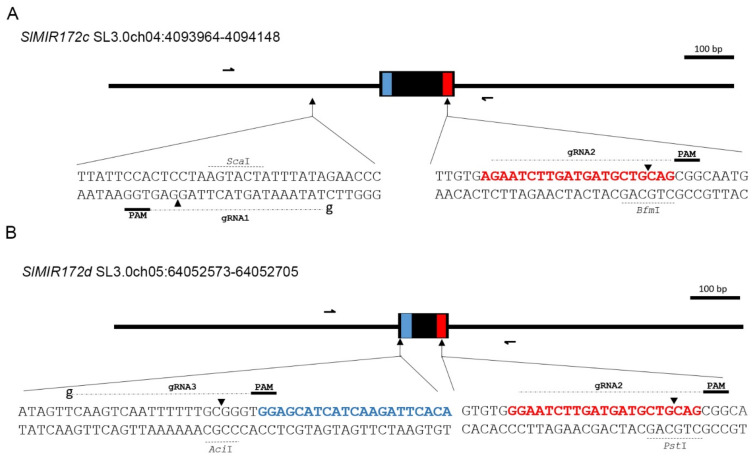
CRISPR/Cas9 targeting of *SlMIR172c* and *SlMIR172d*. (**A**,**B**) Schematic illustrations of the *SlMIR172c* (**A**) and *SlMIR172d* (**B**) gene loci. The locations of corresponding pre-miRNAs in the tomato genome are indicated. Black, red and blue boxes indicate the pre-miR172, mature sly-miR172 and miR172*, respectively. Half arrows indicate primers used for genotyping. The sequences that were targeted by Cas9 are indicated in the expanded regions. In each region, the sly-miR172 and sly-miR172* sequences are colored in red and blue, respectively, the gRNA-targeted sequences are indicated, Cas9 cleavage sites are indicated by arrowheads, the PAM sequences and restriction enzyme sites that were utilized for genotyping are marked.

**Figure A2:**
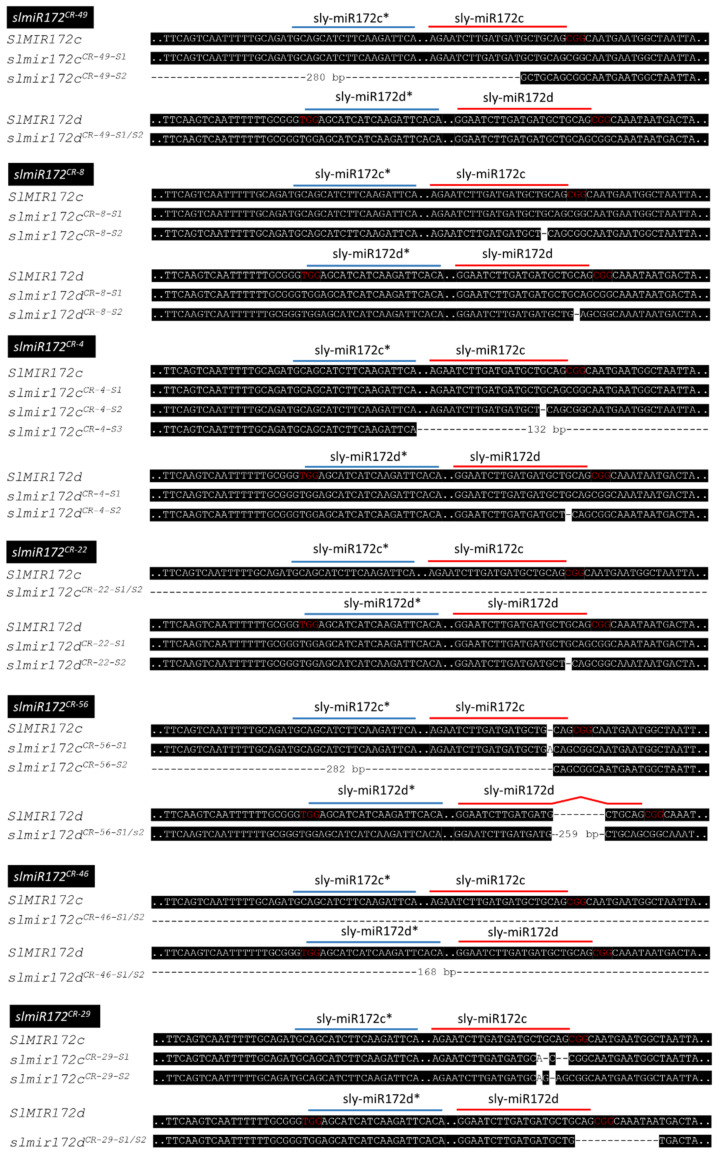
Multiple sequence alignments between wild-type *SlMIR172c* and *SlMIR172d* and respective CRISPR alleles (*slmir172c-d^CR^*) that were recovered from the primary transformants. Transgenic plant number (CR-#) and sequenced mutant alleles (S) are indicated. The respective sly-miR172 and sly-miR172* sequences are indicated and the PAM sequences are colored in red. Numbers indicate the size of deletion in base pairs.

To elucidate the functions of sly-miR172c and sly-miR172d in flower development, and since loss-of-function mutants are not available, CRISPR/Cas9 technology was used to mutate their respective genes. Three guide RNAs (gRNAs) were designed, with gRNA1 and gRNA3 targeting specific sequences upstream to the miRNA* sequence in *SlMIR172c* and *SlMIR172d*, respectively, and gRNA2 that target the 3′ end of the mature miRNA in both (Figure 2A,B). This approach was expected to eliminate the mature miRNA sequence specifically from *SlMIR172c* and *SlMIR172d*, thereby generating loss-of-function mutants. Genotyping by PCR, followed by sequencing of independent primary transgenic plants (T_0_) revealed plants with wild-type alleles, as well as plants harboring large or small indels in one or both targeted *SlMIR172* genes (Figure A2). Mutant alleles (*slmir172c-d^CR^*) containing significant deletions of corresponding pre-miRNA sequences were considered as null alleles. This category included mutant alleles with deletions of both mature and star sequences (*slmir172c^CR-22-S1/S2^*, *slmir172c^CR-46-S1/S2^*, *slmir172d^CR-46-S1/S2^*), deletion of the mature miRNA sequence (*slmir172c^CR-4-S3^*), or the mature miRNA seed sequence (*slmir172c^CR-49-S2^*, *slmir172c^CR-56-S2^*). A mutant allele with insertion of 259 bp within the sly-miR172d sequence was also considered as null allele (*slmir172d^CR-56-S1/S2^*). On the other hand, mutant alleles containing small indels within the 3′ end of the mature miRNA sequence, which is less critical for its function, were considered as hypomorphic alleles. This category included weak alleles with 1 bp indels, which was not expected to abolish the miRNA hybridization to its target sequences (*slmir172c^CR-8-S2^*, *slmir172d^CR-8-S2^*, *slmir172d^CR-22-S2^*, *slmir172c^CR-4-S2^*, *slmir172d^CR-4-S2^*, *slmir172c^CR-56-S1^*), and strong alleles with deletions larger than 1 bp, which are expected to significantly interfere with the hybridization of the miRNA to its target sequence (*slmir172c^CR-29-S1^*, *slmir172c^CR-29-S2^*, *slmir172d^CR-29-S1/S2^*).

### 2.3. SlMIR172d Is Required for Proper Development of All Flower Whorls

**Figure 3:**
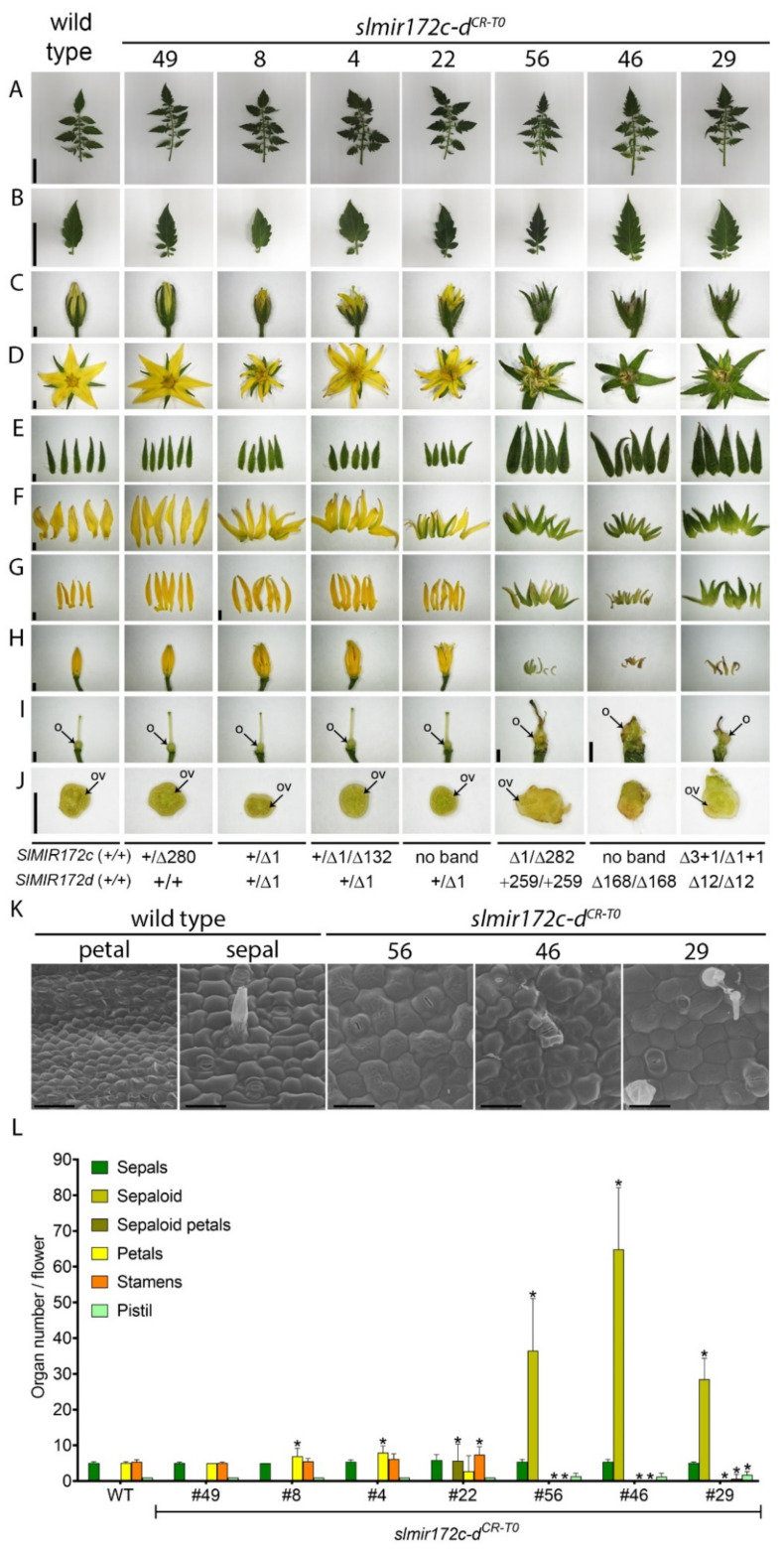
Leaf and flower phenotypes of *slmir172c-d^CR-T0^* plants. (**A**–**J**) Representative pictures of wild type and indicated mutant plants fully expanded leaves (**A**), their terminal leaflets (**B**), stage 18 flower buds (**C**), anthesis flowers (**D**), their sepals (**E**), their petals or sepaloid organs (**F**), their stamens or sepaloid organs (**G**), their anther cone or sepaloid organs (**H**), their pistils (**I**) and manual cross sections of respective ovaries (**J**). O: ovary; OV: ovule. Scale bars = 5 cm for **A**,**B**; 2 mm for **C**–**J**. (**K**) Scanning electron micrographs of the adaxial side of wild-type petals and sepals and indicated *slmir172c-d^CR-T0^* plant sepal-like organs. Scale bars = 50 μm. (**L**) Quantitation of flower organs in wild type and indicated mutant plants. Error bars indicate ± standard deviation (*n* =10). Asterisks indicate significant difference (*p* ≤ 0.01) compared with wild type as determined by Student’s *t*-test.

**Figure 4:**
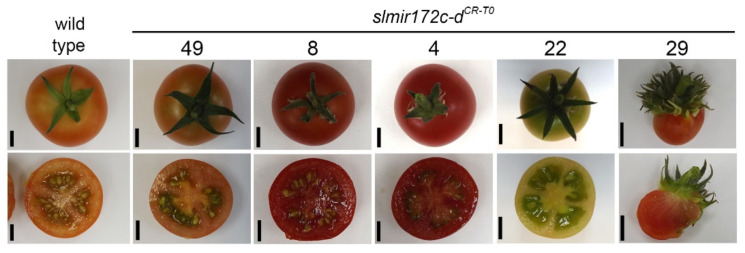
Fruit phenotypes of *slmiR172c-d^CR-T0^* plants. Representative pictures of indicated wild type and mutant whole fruits and their manual cross-sections. Scale bars = 1 cm.

During vegetative development, the leaves of the *slmir172c-d^CR-T0^* mutants did not differ from wild-type leaves suggesting that sly-miR172c and sly-miR172d are not involved in their development (Figure 3A,B). In contrast, during flowering, abnormalities with graded severity in flower morphology were observed (Figure 3C–J), and accordingly the *slmir172c-d^CR-T0^* mutant plants were classified into three groups. The first group included plant *slmir172c^CR-^*^49^ that showed no flower abnormal phenotypes (Figure 3C–J,L), and later-on developed normal looking seeded fruits (Figure 4). The second group of plants included *slmir172c-d^CR-^*^8^, *slmir172c-d^CR-^*^4^ and *slmir172c-d^CR-^*^22^ that produced flowers with normal looking sepals and pistils. However, their petals and stamens were progressively greener rather than completely yellow, suggesting different degrees of acquiring a sepal identity. In addition, they produced supernumerary petals (*slmir172c-d^CR-^*^4^, *slmir172c-d^CR-^*^8^) or supernumerary sepaloid petals and stamens (*slmir172c-d^CR-^*^22^). Moreover, their stamens tend to separate from each other forming a loose cone. The extent of sepal, stamen and anther cone phenotypic severity varied so that *slmir172c-d^CR-^*^22^ > *slmir172c-d^CR-^*^4^ and *slmir172c-d^CR-^*^8^ (Figure 3F–H,L). Nevertheless, their flower’s pistil were not different from wild-type pistils (Figure 3I) and accordingly, these mutants were able to set normal looking seeded fruits (Figure 4). The third group of *slmir172c-d^CR-T0^* mutants included *slmir172c-d^CR-^*^56^, *slmir172c-d^CR-^*^46^ and *slmir172c-d^CR-^*^29^ that exhibited flowers showing high degree of morphological abnormalities. The outer whorl of the flowers of these mutants contained normal number of larger than wild type sepals, and their second and third whorls contained together between 30 and 70 sepal-like organs, instead of 5 petals and 5 stamens, respectively, (Figure 3D–H,L). Similar to wild-type sepals and unlike wild-type petals, the epidermis of the sepal-like organs was composed of pavement cells inlaid with stomata, thus confirming their sepaloid identity (Figure 3K). Moreover, the pistil of the third group mutants was composed of degenerate style and deformed infertile ovary, which could not set fruit (Figure 3I,J). In the case of *slmir172c-d^CR-^*^29^, occasionally its abnormal ovary expanded to form small parthenocarpic fruit-like organ (Figure 4).

**Figure 5:**
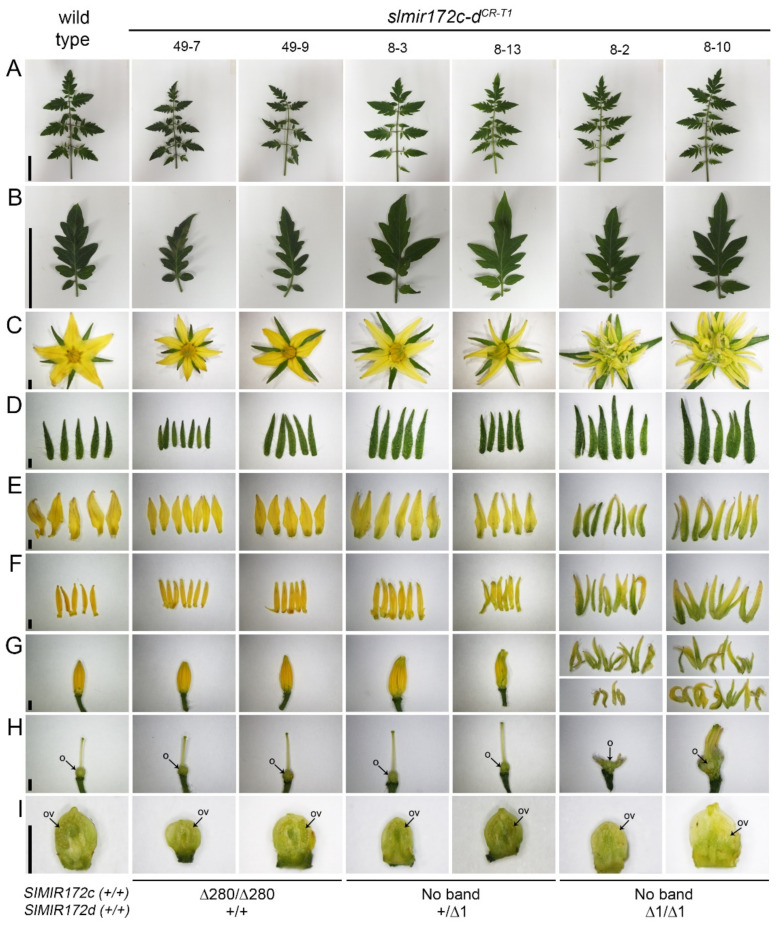
Leaf and flower phenotypes of *slmir172c-d^CR-T1^* plants. (**A**–**I**) Representative pictures of wild type and indicated mutant plants fully expanded leaves (**A**), their terminal leaflets (**B**), anthesis flowers (**C**), their sepals (**D**), their petals or sepaloid organs (**E**), their stamens or sepaloid organs (**F**) their anther cone or sepaloid organs (**G**), their pistils (**H**) and manual longitudinal section of corresponding ovaries (**I**). O: ovary; OV: ovule. Scale bars = 5 cm for (**A**,**B**); 2 mm for (**C**–**I**). The genotype of each plant is marked at the bottom.

We noticed that the severe flower phenotype of the group III mutants was associated with null (*slmir172c-d^CR-^*^56^, *slmir172c-d^CR-^*^46^) or strong (*slmir172c-d^CR-^*^29^) *slmir172d^CR^* mutant alleles. On the other hand, the relatively milder flower phenotypes of groups II mutants were associated with heterozygous hypomorphic *slmir172d^CR^* mutant alleles (*slmir172c-d^CR-^*^4^, *slmir172c-d^CR-^*^8^, *slmir172c-d^CR-^*^22^). Moreover, in the absence of *slmir172d^CR^* mutant alleles (*slmir172c^CR-^*^49^) developed flowers were not different from wild type. We further analyzed the progeny of *slmir172c^CR-^*^49^ and *slmir172c-d^CR-^*^8^ mutant plants. Figure 5 shows representative progeny plants that are homozygote for *slmir172c^CR^* null allele and either wild type, heterozygous or homozygous for *slmir172d^CR^* hypomorphic allele. Plants that harbored wild type *SlMIR172d* alleles (*slmir172c^CR-^*^49-7^, *slmir172c^CR-^*^49-9^) developed normal looking leaves and flowers. However, an atypical green pigmentation was noticed in the basal part of the flower petals. Similar but stronger and more broad green pigmentation of the flower petals was observed in the progeny plants harboring heterozygote hypomorphic *slmir172d^CR^* allele (*slmir172c-d^CR-^*^8-3^, *slmir172c-d^CR-^*^8-13^). Moreover, their stamens were also greenish and formed deformed anther cone (Figure 5E–G). More severe floral organ phenotypes were observed in progeny mutant plants that were homozygous for *slmir172d^CR^* hypomorphic allele (*slmir172c-d^CR-^*^8-2^, *slmir172c-d^CR-^*^8-10^). Their flowers sepals were larger than wild-type, and they developed supernumerary sepaloids in account of petals and stamens and severely distorted pistils containing ovaries, which did not set fruits (Figure 5C–I). These flower phenotypes were highly reminiscent that of category III T_0_ flower phenotypes (*slmir172c-d^CR-^*^56^, *slmir172c-d^CR-^*^46^, *slmir172c-d^CR-^*^29^; Figure 3D–J), except that the sepaloids were less greenish (compare Figure 3F–H to Figure 5F,G). Taken together, the observed mutant flower phenotypes indicated the predominate role of *SlMIR172d* over *SlMIR172c* in floral organ development.

### 2.4. Sly-miR172c and Sly-miR172d Target mRNAs

**Figure 6:**
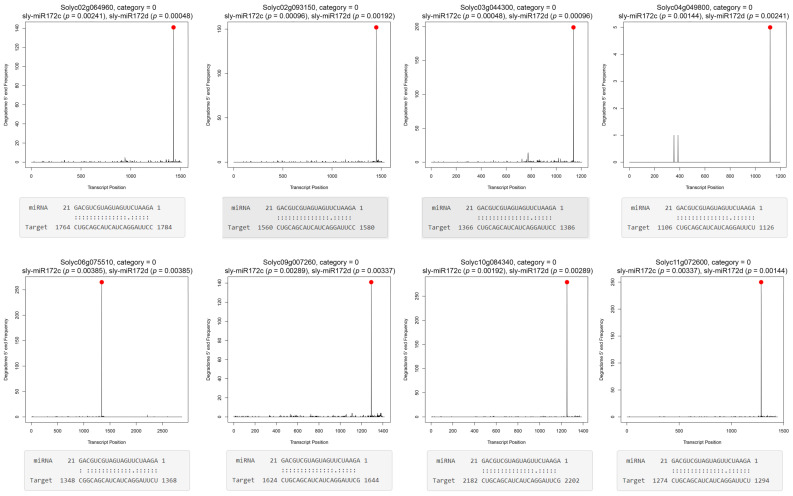
mRNAs predicted to be guided to cleavage by sly-miR172c and sly-miR172d in the flower. Target plots of sly-miR172c and sly-miR172d target mRNAs. The plots are identical for both miRNAs except for the indicated *p*-value. Peaks of reads that their 5′ end is at the predicted cleavage site of sly-miR172c and sly-miR172d are indicated by red circles. Below each plot, Watson-Crick pairing between indicated target sequence and sly-miR172c as predicted by psRNATarget. Similar parings were predicted also for sly-miR172d except for its 5′ nucleotide that is G.

Plant miRNAs show high complementarity to most of their known targets throughout their length [35], and accordingly, their primary mode of action is target cleavage (‘slicing’) [36]. However, miRNA-mediated inhibition of target translation was also demonstrated, including for miR172 [7], and also requires good complementarity between the miRNA and its mRNA target [37]. Therefore, to reveal sly-miR172c and sly-miR172d targets in tomato, we first computationally predicted their complementary mRNA targets by psRNATarget [38]. To reduce false positives, we included only targets that were predicted with a maximum expectation value of ≤3.0, resulting in the prediction of 36 targets for both sly-miR172c and sly-miR172d (Table S3a). To gain further support for our target prediction we next analyzed degradome data from tomato flowers that is available at the SRA database (SRR057484; [39]). Downloaded sequences underwent initial processing and then analyzed by CleaveLand 4.0 [40] using as quarries mature sly-miR172c and sly-miR172d sequences. To reduce false positives in CleaveLand prediction, even on the expense of true putative targets, we considered only statistically significant cleavage site hits at category 0, which is defined as a single maximum of sequence tags at the transcript miRNA cleavage site. A total of eight identical mRNAs were found to be targeted by both sly-miR172c and sly-miR172d at categories 0 (*p* ≤ 0.01) (Table S3b,c). These sly-miR172c and sly-miR172d targets were also predicted by psRNATarget (Table S3a), and previously identified as targets of sly-miR172a and sly-miR172b, through analysis of tomato fruits degradome [41], further corroborating their authenticity. The “target plots” (t-plots) of these targets are shown in Figure 6.

**Figure 7:**
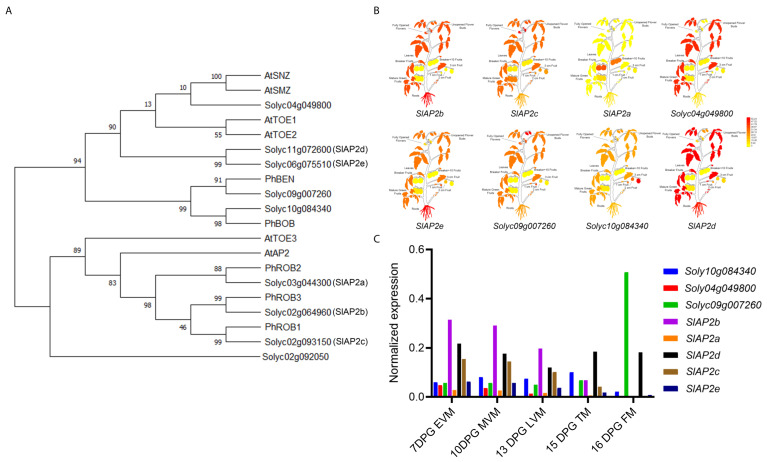
Characterization of *SlAP2* genes predicted to be targeted by sly-miR172c and sly-miR172d. (**A**) A rooted phylogenetic tree based on the protein sequences of *Arabidopsis* (At), petunia (Ph) and tomato eu-AP2 transcription factors. The Solyc02g092050 protein sequence served as an outgroup. The tree was constructed using FastTree v2.1.8 with default parameters [34]. (**B**) Tissue based expression profiles of indicated genes in tomato CV Heinz. Yellow and red indicate, weak and strong expression, respectively. Expression data was retrieved from the ePLANT database (http://bar.utoronto.ca/eplant_tomato/, accessed on 1 January 2021). (**C**) Normalized expression levels of indicated genes in vegetative and flower meristems. Expression data was retrieved from the TomExpress database (http://tomexpress.toulouse.inra.fr, accessed on 1 January 2021). EVM, early vegetative meristem; MVM, mature vegetative meristem; LVM, late vegetative meristem; TM, transition meristem; FM, flower meristem.

Annotation of the proteins encoded by sly-miR172c and sly-miR172d target mRNAs showed that they belong to the euAP2 transcription factor family. Phylogenetic analysis of these proteins along with the *Arabidopsis* and petunia euAP2 transcription factors suggested that predicted target proteins cluster into two well-supported clades the TOE and AP2 types. While SlAP2a, SlAP2b SlAP2c proteins seem to belong to the AP2 class, Solyc04g049800, Solyc11g072600 (SlAP2d), Solyc06g075510 (SlAP2e), Solyc09g007260 and Solyc10g084340 are TOE-type proteins (Figure 7A). The phylogenetic analysis indicates that Solyc09g007260 is closely related to PhBEN and Solyc10g084340 is closely related to petunia BROTHER OF BEN (PhBOB). The AP2-type proteins: SlAP2a, SlAP2b and SlAP2c are closely related to PhROB2, PhROB3 and PhROB1 respectively, (Figure 7A). We further evaluated the expression patterns of sly-miR172c and sly-miR172d targeted *euAP2* genes from publicly available data. This analysis shows that *Solyc09g007260* is the most abundant *euAP2* gene in flower buds and flower meristem (FM) and *SlAP2d* is the second abundant gene in the FM (Figure 7B,C). The expression levels of *SlAP2b* and *SlAP2c* are relatively high in vegetative meristems but relatively low in the FM. The latter is most abundant in open flowers, while *Solyc10g084340* and *SlAP2a* are relatively abundant in developing and ripening fruits, respectively, but low in the FM (Figure 7B,C). These results suggest that certain sly-miR172c/d-targeted *euAP2* play roles in tomato flower and fruit development and are probably differentially regulated by sly-miR172 in respective tissues. 

## 3. Discussion

The traditional approaches to study miRNA functions such as target mimicry and expression of a miRNA-resistant target mRNA are more suitable to study the generic function of a miRNA family rather than the function of specific miRNA family member. However, recently, the inactivation of specific *MIR* genes using CRISPR/Cas9 has proven useful to decipher the roles of individual miRNA family members [17,18,28]. Here, to identify the sly-miR172 members that are regulating flower development in tomato, several null and hypomorphic mutants were generated in *SlMIR172c* and *SlMIR172d*, which code for the predominant miR172 members in flower buds. Inactivation of *SlMIR172c* had little effect on floral organ development as best demonstrated by the near wild-type phenotype of the *SlMIR172c* specific mutant *slmir172c^CR-49^* flowers (Figure 5). Nevertheless, floral organ abnormalities in heterozygous *slmir172d^CR^* backgrounds were intensified in the absence of sly-miR172c activity (Figure 3). These observations suggest that *SlMIR172c* contribution to the overall miR172 regulation of flower development is relatively minor and likely redundant to *SlMIR172d*. Although a specific mutant in *SlMIR172d* was not isolated in this study, analysis of the available CRISPR mutants showed that a mutation in *SlMIR172d*, even in the heterozygous state, was always associated with flower organs abnormalities (Figure 3 and Figure 5). This strongly suggests that *SlMIR172d* is required for floral organ development and most likely plays a more significant role than *SlMIR172c* in this process.

A notable flower phenotype of plants homozygous for *slmir172d^CR^* allele was the conversion of the petals and stamens into sepaloids. While loss of sly-miR172d activity led to their complete transformation into sepal-like organs (*slmir172c-d^CR-^*^46^), a partial loss of sly-miR172d activity caused an incomplete conversion of their identity (*slmir172^CR-^*^8-2^, *slmir172^CR-8-10^*). Previously, overexpression of sly-miR172a induced the conversion of sepals into petals in a dose-dependent manner [30]. Together, these findings suggest that sly-miR172d regulates in a quantitative manner the identity of petals and stamens. According to the ABC model in *Arabidopsis* [19,20,21,23,25], the development of sepaloids in the second and third whorls, as observed in the *slmir172d^CR^* mutant flowers, can be explained by expansion of the A-function activity into the third floral whorl and in parallel reduction of the B-function activity in the second and third whorls. Eight *euAP2s*, with five genes that belongs to TOE-type clade and three to AP2-type clade, were predicted to undergo sly-miR172c and sly-miR172d-guided cleavage in flowers. While in *Arabidopsis*, the A-function is fulfilled by AP2-type proteins and not by TOE-type proteins, in *Solanaceae* species such as petunia, the A-function is fulfilled by both [26]. Thus, each of the predicted targets of sly-miR172c and sly-miR172d may function as an A-class genes. In contrast to *Arabidopsis*, in which B-function genes are not repressed by A-function genes, in petunia, the A-function genes encoded by euAP2s are a major B-function repressors in the first floral whorl [26]. Our results strongly support that similar mechanism works in tomato so that reduced sly-miR172c-d activity eventually leads to reduced activity of B-function genes, most probably due to increased transcription and translation of sly-miR172c-d targets.

In *Arabidopsis*, expression of miR172-resistant *AP2* resulted in indeterminate FM that caused the production of flowers with excess organs [24]. Similarly, tomato plants containing *slmir172d^CR^* allele developed excess organs in the second and third whorls of their flowers, a phenotype which was more pronounced in homozygous mutants. These raise the possibility that upregulation of sly-miR172d-targets in *slmir172d^CR^* mutants, reduced the determinacy of the FM. In tomato, it was demonstrated that silencing of the C-function gene TAG1 resulted in indeterminacy of the flower meristem [42]. Consistent with the ABC model in *Arabidopsis* [11,21,23,24,25], this suggests that in tomato, the A-function genes targeted by sly-miR172d, likely function as suppressors of the C-function activity, required for flower meristem determinacy. However, sly-miR172d loss-of-activity did not completely abolish determinacy, since the fourth whorl organs were present in all mutant flowers, albeit with abnormal morphology. It was shown that in petunia, the C-activity is restricted by the euAP2 A-function gene *PhBEN* and mainly by the *MIR169* gene *BLIND*, which negatively regulate *NF-YA* [26,27]. A similar parallel suppression of C-function by *SlMIR169* and euAP2s in tomato can explain why eliminating sly-miR172d activity reduced and not abolished FM determinacy.

In *Arabidopsis* CRISPR-meditated mutagenesis of miR172 revealed its roles in phase transition, flowering time, and floral organ development [17,18]. In this study, we deciphered the functions of *SlMIR172c* and *SlMIR172d* in flower development. Still, sly-miR172 is expressed during fruit development and are predicted to guide the cleavage of *SlAP2a*, which is involved in fruit ripening [30,43,44,45]. Future studies will focus on functional analysis of the remaining five tomato *SlMIR172* genes, with the hope to expand the knowledge on the roles of this important miRNA family in plants.

## 4. Materials and Methods

### 4.1. Plant Material and Growth Conditions

Tomato cv. MP-1 plants [46] were grown in 400-mL pots under greenhouse conditions with temperatures ranging between 15 and 30 °C in a tuff-peat mix with nutrients. Germination and seedling growth took place in a growth chamber under a 16-h light, 8-h dark photoperiod at a constant temperature of 24 °C. At around one month post germination seedlings were transferred to the greenhouse. For in vitro culture, seeds were surface-sterilized by treatment with 2% hypochlorite solution for 5 min. After rinsing three times with sterile distilled water, seeds were sown on Murashige and Skoog culture medium containing 3% (*w*/*v*) sucrose and 1% (*w*/*v*) phytoagar (Sigma, Rehovot, Israel), pH 5.8.

### 4.2. Plasmid Construction

For the CRISPR/Cas9 mediated mutagenesis of *SlMIR172c* and *SlMIR172d* genes, an identical common gRNA targeting their mature sly-miR172 and two additional gene-specific gRNAs targeting the sequences upstream to their mature miRNA (The gRNAs sequences are indicated in Figure 2A,B) were designed using the CHOPCHOP tool (http://chopchop.cbu.uib.no, accessed on 1 January 2021). Each gRNA sequence was incorporated in silico into sgRNA consisting of its 20 bp followed by a 76 bp generic scaffold and a 7xT Polymerase III terminator sequence. Then a construct containing the three sgRNAs in tandem, each under the control of the synthetic Arabidopsis U6 promoter, delimited by 5′-*Mlu*I and 3′-*Hind*III, was artificially synthesized (GENEWIZ, South Plainfield, NJ, USA) and cloned into pUC57. The pUC57-sgRNAs was digested with *Mlu*I and *Hind*III and the released sgRNAs fragment was ligated into the compatible sites of the pRCS binary vector alongside the plant codon-optimized version of Cas9 [47] expressed under the constitutive CaMV 35S promoter to generate pRCS-35S::Cas9-SlMIR172c-d.

### 4.3. Tomato Transformation

The binary plasmid pRCS-35S::Cas9-SlMIR172c-d was transformed into tomato cultivar MP-1 by co-cultivation of cotyledons derived from 14-day-old seedlings with *Agrobacterium tumefaciens* strain EHA105 as described previously [46], followed by regeneration on selective 100 mg/l kanamycin-containing media, where only transgenic seedlings developed a branched root system. The transgenic status of the kanamycin resistant seedlings was further validated by genomic DNA PCR with the primer pair 5′-CGACAATCTGATCCAAGCTCA-3′ and 5′-GACACTGACGGCTTTATGCC-3′ to detect the presence of the *Cas9* transgene.

### 4.4. Isolation of SlMIR172c and SlMIR172d CRISPR Mutants

To isolate CRISPR mutants in *SlMIR172c* and *SlMIR172d* genes, the primary transformants and their progeny if available were screened by genomic DNA PCR with specific primers flanking the gRNA targeted sequences (5′-CATCAGTCATCAAGTCA TCCAAAG-3′ and 5′-ATTTGAAAGGGGTAAGTTTTCTT-3′ for *SlMIR172c* and 5′-TTTCC CATTTCTCCCTTCCT-3′ and 5′-TTTCCGTTGAAGAAAGTTTGG-3′ for *SlMIR172d*) followed by agarose gel electrophoresis, to detect amplicons with significantly different size which indicated mutant alleles containing relatively large indels. These amplicons were directly sequenced to determine the nature of the mutations. If the PCR amplicons were of approximately the wild-type expected size they were further cloned into pGEM-T (Promega, Madison, WI, USA), and five colonies were sequenced per amplicon to detect potential mutant alleles.

### 4.5. Scanning Electron Microscopy (SEM)

For SEM, floral organs were harvested from anthesis flowers and placed in FAA (3.7% formaldehyde, 5% acetic acid, 50% EtOH) solution until use. Then the FAA solution was removed and samples were dehydrated in an increasing gradient of ethanol (up to 100%), critical-point-dried, mounted on a copper plate and palladium-coated. Samples were viewed in a JCM-6000 SEM microscope (JEOL, Akishima, Japan).

## Data Availability

Not applicable.

## Supplementary Materials

The following are available online at https://www.mdpi.com/article/10.3390/ijms22094659/s1.

## Abbreviations

AG	AGAMOUS
AP	APETALA
Cas9	CRISPR associated protein9
CRISPR	Clustered Regularly Interspaced Short Palindromic Repeats
ERF	Ethylene Responsive Factor
gRNA	guide RNA
miRNA	microRNA
PI	PISTILLATA
SNZ	SCHNARCHZAPFEN
SMZ	SCHLAFMUTZE
SPL	SQUAMOSA PROMOTER BINDING PROTEIN-LIKE
TOE	TARGET OF EAT
